# Optimized nanostructured In_2_O_3_ gas sensors: harnessing annealing-induced defects and oxygen vacancies for ultra-sensitive and selective H_2_S detection at trace levels†

**DOI:** 10.1039/d5ra01394a

**Published:** 2025-05-19

**Authors:** Tanya Sood, Ramseena Thundiyil, Anusha, Saikat Chattopadhyay, P. Poornesh

**Affiliations:** a Department of Physics, Manipal Institute of Technology, Manipal Academy of Higher Education Manipal 576104 India poornesh.p@manipal.edu poorneshp@gmail.com; b Department of Physics, School of Basic Sciences, Manipal University Jaipur Jaipur 303007 India

## Abstract

Achieving selectivity and high sensitivity for specific analytes at trace levels remains a significant challenge for chemiresistive gas sensors. In this study, nanostructured indium oxide (In_2_O_3_) gas sensors were synthesized *via* spin coating for detection of hydrogen sulphide (H_2_S) gas at trace levels. The influence of annealing temperature on the gas sensing performance for the deposited nanostructured gas sensors was systematically investigated. The sensor annealed at 350 °C exhibited outstanding performance, with rapid response time of (17 ± 1) seconds for H_2_S gas concentrations of 4 ppm, at an optimal operating temperature of 250 °C. Additionally, it achieved an exceptional sensing response of (36.52 ± 2.02)% and (97.89 ± 0.08)% for 0.5 ppm and 4 ppm H_2_S gas respectively. The remarkable sensing performance is attributed to the presence of structural defects, voids and oxygen vacancies, which enhance gas adsorption and reactivity. These findings demonstrate that In_2_O_3_ nanostructured gas sensors are highly effective for the reliable detection and monitoring of H_2_S gas in practical applications.

## Abstract 2

In_2_O_3_ nanostructured films exhibit superior sensitivity, selectivity, and stability towards H_2_S sensing.

## Graphical abstract

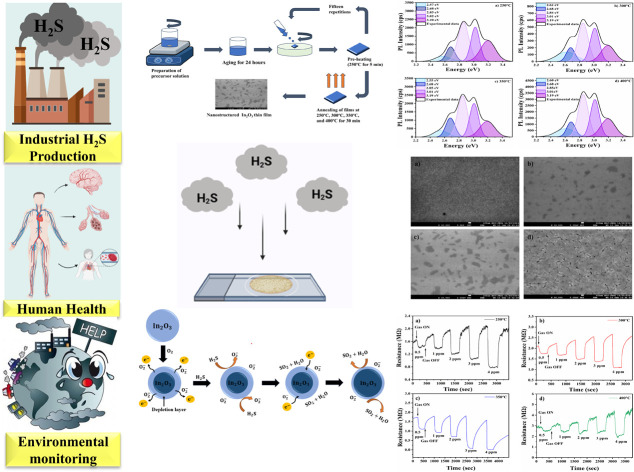

## 1. Introduction

The rapid expansion of industry, coupled with a growing global population, has led to a significant decline in air quality due to the increasing release of harmful pollutants. Among these pollutants, particulate matter (PM2.5 and PM10), nitrogen dioxide (NO_2_), carbon monoxide (CO), ammonia (NH_3_), sulfur dioxide (SO_2_), and hydrogen sulfide (H_2_S) pose serious threats to both human health and the environment.^1^ H_2_S is highly toxic even at low concentrations, making it a critical target for air quality monitoring systems. It is emitted from both natural and anthropogenic sources, including sewage treatment plants, volcanic activity, oil refining, and natural gas processing.^2^ Due to its extreme toxicity, both acute and chronic exposure can lead to severe respiratory distress, neurological impairment, and, in high concentrations, fatalities. Recognizing these dangers, regulatory agencies such as the Occupational Safety and Health Administration (OSHA) in the United States have set strict exposure limits. OSHA enforces a short-term exposure limit (STEL) of 15 ppm for a 10 minutes period, while the American Conference of Governmental Industrial Hygienists (ACGIH) has established a threshold limit value (TLV-TWA) of 1 ppm for an 8 hours workday. These regulations underscore the urgent need for reliable H_2_S detection systems.^3,4^

To mitigate the risks associated with H_2_S exposure, accurate and continuous monitoring is essential. Conventional gas detection systems, such as electrochemical and optical sensors, have been widely used but suffer from limited lifespan, high power consumption, and susceptibility to environmental interference.^5^ Therefore, there is an increasing demand for lightweight, compact, energy-efficient, and highly selective gas sensors that can operate reliably in various environments, including industrial facilities, confined spaces, and urban settings.^6^ Sensors must also exhibit high sensitivity for low-concentration detection, long-term stability, and cost-effectiveness to ensure widespread deployment. Developing such advanced sensors is crucial for the early detection of H_2_S, allowing preventive actions before it reaches hazardous levels.^7^

Among various gas sensing materials, metal oxide semiconductors (MOS) have emerged as promising candidates due to their low-cost fabrication, high sensitivity, and fast response times. MOS materials such as WO_3_,^8^ SnO_2_,^9^ In_2_O_3_,^10^ Fe_2_O_3_,^11^ CuO,^12^ ZnO,^13^ and NiO^14^ are widely used for gas sensing applications, benefiting from their excellent thermal stability and well-understood sensing mechanisms. Among these metal oxides, In_2_O_3_ stands out as a highly promising gas sensing material due to its exceptional electrical conductivity, chemical stability, tuneable surface properties, and ability to detect a wide range of toxic gases, including NH_3_,^15^ CO,^16^ H_2_S,^17,18^ and NO_2_.^19^ Its n-type semiconducting behaviour, with a direct band gap of 3.6 eV and an indirect band gap of 2.6 eV, enables efficient charge carrier transport, making it suitable for gas sensing applications as well as its use in transparent conducting oxides (TCOs),^20^ optoelectronics, light-emitting diodes (LEDs), and electrochromic devices.^21^ Additionally, its ability to absorb and transmit visible light contributes to its applicability in electrical switching and other advanced electronic applications.^22^ The presence of oxygen vacancies enhances its sensing performance, improving response time and sensitivity.^23^ Despite these advantages, challenges remain in optimizing sensor selectivity, response stability, and large-area fabrication, necessitating further investigation into novel deposition techniques and material modifications. Extensive research has been conducted to develop In_2_O_3_ based gas sensors, with a focus on improving their sensitivity, selectivity, and stability.^24^ Various deposition techniques are employed to fabricate In_2_O_3_ thin films, each offering unique advantages in terms of film quality, scalability, and cost-effectiveness. These include physical methods such as thermal evaporation, e-beam evaporation, sputtering, pulsed laser deposition (PLD)^25^ and chemical techniques such as spray pyrolysis,^26^ thermal hydrolysis,^27^ dip coating, electrospinning, sol–gel,^28^ spin coating,^29^ solvothermal synthesis, and hydrothermal processing.^30^ Among these, spin coating is widely recognized as an efficient technique for depositing uniform thin films over large areas due to its precise control over chemical composition, high purity, reproducibility, and cost efficiency.^29^

The gas sensing properties of In_2_O_3_ thin films are heavily influenced by film morphology (nanorods, nanosheets, nanoflowers) and microstructure, both of which are determined by the choice of deposition method, processing conditions, and subsequent annealing treatments. Annealing is a crucial post-deposition step that enhances crystallinity, eliminates structural defects, improves film uniformity, and increases grain size, thereby significantly impacting gas adsorption and desorption kinetics.^31^ Moreover, annealing leads to an increase in oxygen vacancies, which act as electron donors, improving charge transport and overall sensor performance. These modifications collectively contribute to higher sensitivity, faster response and recovery times, and improved long-term stability, making annealed In_2_O_3_ an excellent candidate for gas detection applications. Several studies have demonstrated the significant influence of annealing temperature on gas sensing performance. Ravikumar T. *et al.* investigated ZnFe_3_O_4_ films deposited *via* spray pyrolysis, showing a fivefold increase in NH_3_ sensing response upon annealing at 400 °C, compared to the as-deposited film.^32^ Similarly, Sarf F. *et al.* synthesized α-Fe_2_O_3_ thin films *via* spin coating and observed that annealing at 600 °C yielded a 10% enhancement in CO sensing response at ambient temperature, attributing the improvement to better crystallization and increased porosity.^33^ Additionally, Gupta *et al.* employed the sol–gel method to synthesize NiO powders and reported a 244.4% increase in LPG gas sensing response after annealing at 500 °C, demonstrating that optimized annealing conditions significantly enhance sensitivity and selectivity in metal oxide gas sensors.^34^

Building upon these findings, this study systematically investigates the effect of annealing temperature on the structural, morphological, optical, and H_2_S gas sensing characteristics of spin-coated In_2_O_3_ thin films. The annealing process plays a pivotal role in modulating the crystallinity, surface roughness, and oxygen vacancy concentration, which directly affect the sensor's performance. By varying the annealing temperature, its impact on film composition, charge carrier dynamics, and gas adsorption sites can be systematically evaluated. This study presents an optimized In_2_O_3_-based chemiresistive gas sensor for highly selective and sensitive H_2_S detection by systematically tuning the annealing temperature (250–400 °C). The results demonstrate that the annealing at 350 °C significantly enhances the sensor response (97.89 ± 0.08%) due to improved crystallinity, surface interactions and oxygen vacancy modulation. The sensor exhibits a low detection limit of 0.5 ppm, rapid response (17 ± 1 s at 4 ppm H_2_S), and excellent repeatability over multiple cycles, ensuring reliability. Additionally, the study evaluates the effect of temperature on sensor performance, revealing that 250 °C is the optimal operating temperature, balancing adsorption and desorption kinetics. These findings reinforce the critical role of annealing in optimizing In_2_O_3_ based gas sensors and underscore the potential of spin-coated In_2_O_3_ thin films as highly effective, selective, and stable H_2_S detectors for real-world applications in environmental monitoring and industrial safety.

## 2. Experimental details

### 2.1. Preparation of In_2_O_3_ nanostructured thin films

Acetone (CH_3_COCH_3_), isopropanol (C_3_H_8_O), 2-methoxyethanol (C_3_H_8_O_2_), and indium(iii) nitrate hydrate [In(NO_3_)_3_·*x*H_2_O] (99.99% pure), were procured from MERCK. Deionized water (DI) with a resistivity of 18.3 MΩ was used exclusively throughout the process. Microscopic glass slides were obtained from Labtech. These chemicals and materials were employed in the preparation of the precursor solution for the spin coating method.

**Fig. 1:**
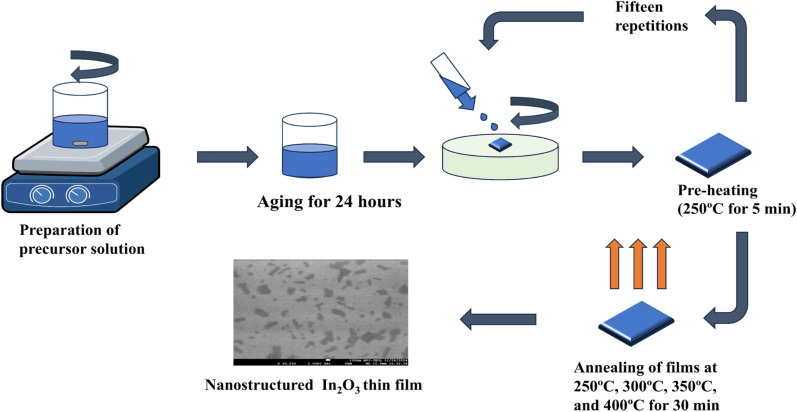
Schematic diagram of preparation of indium oxide nanostructured thin films.

The cleaning process involved sequentially treating the substrates with soap solution, DI water, isopropanol (IPA), and acetone, individually for 10 minutes in an ultrasonic bath. Following the cleaning steps, the substrates underwent air drying with a flow of nitrogen. To further remove organic impurities and minimize surface roughness, ozone treatment was applied for 15 minutes, leading to a more uniform and cleaner surface. 0.2 M of the aqueous precursor solution was then prepared utilizing indium nitrate hydrate, and 2-methoxyethanol. The solution was continuously stirred at 60 °C for 24 hours to achieve a transparent, and homogeneous mixture, followed by aging at room temperature for another 24 hours. The aged precursor solution was coated onto the glass substrate utilizing the spin coating method at a precise speed of 2000 rpm for 30 seconds. The films were subsequently heated at 250 °C for 5 minutes to remove the solvent. This process was performed for 15 times to achieve the desired thickness of 250–300 nm. Subsequently, the films were annealed at temperatures: 250 °C, 300 °C, 350 °C, and 400 °C on a hot plate in ambient air for 30 minutes. Fig. 1 represents the schematic process flow for the preparation of nanostructured In_2_O_3_ thin films at various annealing temperatures.

### 2.2. Characterization of In_2_O_3_ nanostructured thin films

#### 2.2.1. Structural studies

The deposited film's crystal structure was analysed using a Rigaku Miniflex 600 diffractometer equipped with Cu Kα radiation at 40 kV and 15 mA, with a scanning range of 5° to 65° at a rate of 1° min^−1^. Additionally, the composition, impurity levels, phase, and defects of the deposited films were examined using Horiba LabRAM HR Evolution Raman spectroscopy with a 532 nm laser at room temperature. X-ray photoelectron spectroscopy (XPS) was employed to evaluate the oxidation states of the elements present in the deposited films. Measurements were carried out using an AXIS ULTRA instrument equipped with a monochromatic Al Kα X-ray source operating at 14 kV. The C 1s peak at 284.8 eV was used as an internal standard for binding energy calibration.

#### 2.2.2. Morphological and optical studies

Field Emission Scanning Electron Microscopy (FESEM) was utilized to examine the morphology and surface characteristics of the deposited thin films. The analysis was carried out using JEOL JSM-7610F Plus field emission scanning electron microscope, operated at 2 kilovolts, following the sputtering of a thin gold layer onto the samples.

The nanostructured deposited films were analysed for UV-visible light transmission utilizing a 1900i UV-vis spectrophotometer over a wavelength range of 190 to 1100 nm, with glass substrates serving as the reference background (Section 1 of ESI†). Photoluminescence (PL) studies were performed to identify defects within the films using JASCO FP-8300 spectrofluorometer. Measurements were taken from 350 nm to 700 nm at ambient temperature, with an excitation wavelength of 335 nm to acquire the emission spectrum of the thin films.

#### 2.2.3. Gas sensing measurements

**Fig. 2:**
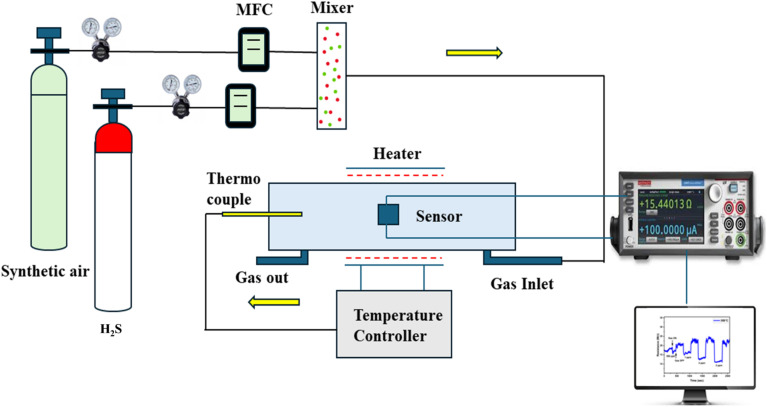
Schematic representation of gas sensing setup.

A uniform layer of conducting silver paste (Sigma Aldrich) was deposited onto the surface of the sensor to establish ohmic interfaces for gas sensing measurements, with each electrode area measuring 5 mm × 5 mm. The sensor was then placed inside a sealed stainless-steel gas sensing chamber equipped with two magnetic probes, which were positioned directly onto the silver electrodes. The sensor was gradually heated to its optimal temperature using an external heating system. Prior to introducing the target gas, high-purity synthetic air (20.9% O_2_ balanced with N_2_, 99.999% purity) was continuously purged into the chamber to stabilize the baseline resistance. The total gas flow rate was maintained at 500 standard cubic centimetres per minute (sccm) using high-precision mass flow controllers (MFCs, Alicat Scientific). After the stabilization of sensor's baseline resistance, the target gases – including H_2_S, NH_3_, CO, SO_2_ and NO_2_ were introduced at concentrations of 0.5, 1, 2, 3 and 4 ppm by dynamically diluting certified gas cylinders with synthetic air in appropriate proportions. All gases were ultra-high purity grade (Chemix specialty gases and equipment), with nitrogen used as the filler gas. The electrical resistance of the sensor was continuously recorded during both gas exposure and recovery using Keithley 2450 Source Meter. The repeatability and long-term stability tests were carried out on the best-performing sensor by comparing the sensor response of a freshly prepared device with that of the sensor stored under ambient conditions and retested after eight months. Fig. 2 illustrates the schematic representation of gas sensing setup. The gas sensing performance was evaluated based on three key parameters: response time, recovery time and sensor response. The response time (*τ*_res_) was determined by measuring the time taken by sensor to reach 90% of its maximum resistance value after the target gas exposure, while recovery time (*τ*_rec_) was determined by measuring the time taken to return to 90% of the baseline resistance once the analyte gas was removed and synthetic air was reintroduced. The sensor response defined as (*S*%) = ((*R*_a_ − *R*_g_)/*R*_a_) × 100,^35^ where *R*_a_ and *R*_g_ are the electrical resistance of the sensors in air and target gas, respectively. The gas responses were calculated using the formulae *S*% = ((*R*_a_ − *R*_g_)/*R*_a_) × 100 for reducing gases and *S*% = ((*R*_g_ − *R*_a_)/*R*_a_) × 100 for oxidizing gases.^36^ All gas sensing measurements were repeated for three consecutive cycles to ensure reproducibility. Statistical significance was determined using one-way ANOVA, with a confidence level of 95% (*p* < 0.05).^37^ Results are presented as mean ± standard deviation, and error bars in plots represent standard deviation calculated from the repeated trials.

## 3. Results and discussions

### 3.1. Structural investigations

#### 3.1.1. XRD analysis

**Fig. 3:**
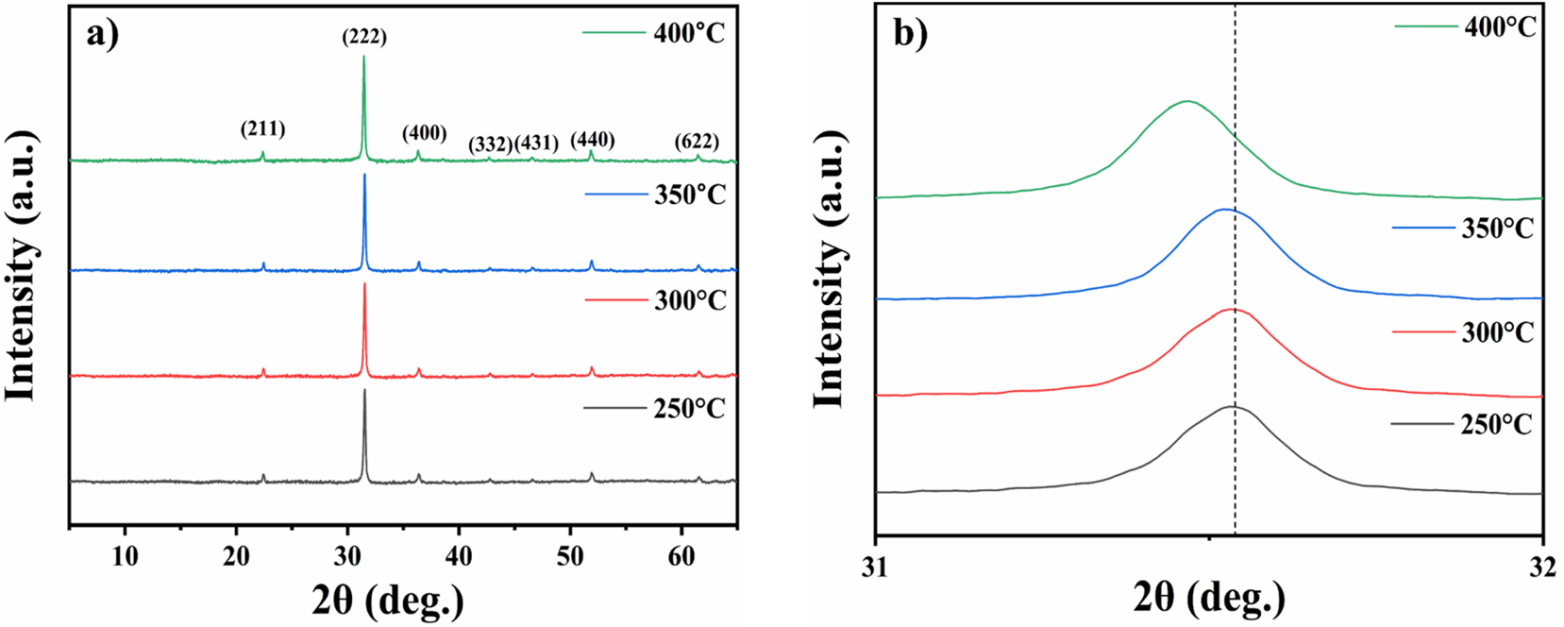
XRD spectra of nanostructured indium oxide thin films for 2*θ* (deg.) (a) 5°–65° and (b) 31°–32°.

Fig. 3(a) shows XRD patterns of deposited In_2_O_3_ thin films at several annealing temperatures. The films characterize polycrystalline nature. The diffraction peaks correspond to cubic structure, with the space group identified as *Ia*3̄, referencing JCPDS card file 06-0416 which corresponds to (211), (222), (400), (332), (431), (440) and (622) planes.^38^ Rising the annealing temperature provides In_2_O_3_ crystallites with sufficient energy to align along the (222) plane. This orientation is preferred because the (222) plane offers the highest atomic packing density and the lowest surface energy.^39^

Additionally, after annealing, all diffraction peaks shifted towards higher 2*θ* angles by 1° when compared to standard diffraction peaks. This suggests that annealing has either reduced the lattice constant or altered the residual strain between the lattice planes, leading to a decrease in the lattice parameter. The similar behaviour is also observed by I. Hotovy *et al.*^40^ For the film annealed at 400 °C, the diffraction peak shifts towards a lower angle, as shown in Fig. 3(b). This demonstrates an orderly lattice expansion.^41^

The crystallite size, microstrain, and dislocation density variations of In_2_O_3_ nanostructured films for various annealing temperatures were calculated and are summarized in Table 1.

**Table 1:** Structural parameters of nanostructured indium oxide thin films

Annealing temperature (°C)	2*θ* (deg.)	Crystallite size (nm)	Strain (×10^−3^)	Dislocation density (×10^14^ lines per m^2^)
250	31.53	43.31 ± 0.16	0.83	5.33
300	31.53	45.01 ± 0.17	0.80	4.93
350	31.53	46.69 ± 0.17	0.77	4.58
400	31.46	44.77 ± 0.16	0.80	4.98

The crystallite size (*D*) was calculated by employing the Scherrer equation, which is expressed as,^42^3.1
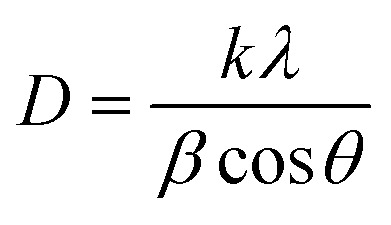
where *k* represents the shape factor, *λ* is the X-ray wavelength, *β* is the full-width half maximum, and *θ* denotes the glancing angle. The crystallite size increases up to 350 °C due to enhanced mobility of the adsorbed atoms on the material surface, promoting cluster formation and subsequent aggregation of small grains. These aggregated grains merge, leading to the formation of larger grains with enhanced crystallinity.^43^

The strain was determined using the formula,^43^3.2
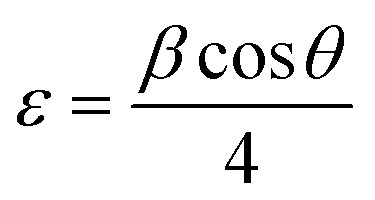


The dislocation density which is stated as the dislocations per unit length and is given by,^43^3.3
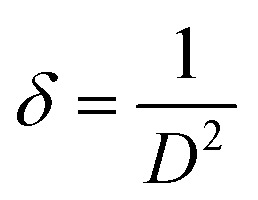


As the annealing temperature rises to 350 °C, the microstrain (*ε*) as well as the dislocation density (*δ*) decrease, resulting in fewer lattice imperfections.^44^ As the annealing temperature increases to 400 °C, In_2_O_3_ bonds break instead of allowing atoms to freely move to their steady sites. This leads to the formation of stress and defects in the film. S. Pandey *et al.* similarly observed this behaviour in the sputtered ZnO films.^45^

#### 3.1.2. Raman spectroscopic analysis

Raman spectroscopy is an important instrument for characterizing metal oxides, as it enables the identification of specific vibrational modes associated with the material's crystal lattice and its structural properties. The measurements were performed utilizing Raman instrument having an excitation wavelength of 532 nm to analyse the nanostructured In_2_O_3_ thin films. The vibrational spectra of the In_2_O_3_ were observed within the range of 100–800 cm^−1^. Based on the previous studies, the cubic phase of In_2_O_3_ is associated with I^3^_a_ space group which is equivalent to T^7^_h_ in Schoenflies notation. The group theory analysis predicts the vibrational modes for I^3^_a_ space group as^46^

3.4 *Γ* = 4A_g_ + 4E_g_ + 14T_g_ + 5A_u_ + 5E_u_ + 16T_u_

where A_g_, E_g_, and T_g_ denote Raman-active vibrational modes, T_u_ represents IR-active vibrations. Both A_u_ and E_u_ are inactive in Raman and IR spectroscopy.

**Fig. 4:**
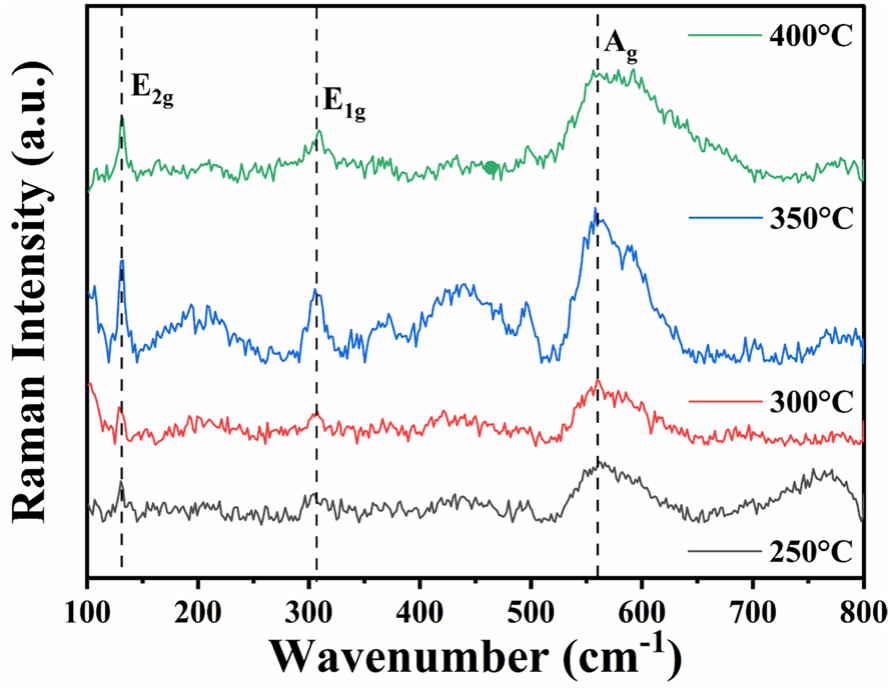
Raman spectra of indium oxide nanostructured films for various annealing temperatures.

Fig. 4 shows the Raman intensity *vs.* wavenumber graph for indium oxide thin films that exhibit E_2g_ mode at 130 cm^−1^, E_1g_ mode at 306 cm^−1^, and A_g_ mode at 561 cm^−1^.^47^

##### 3.1.2.1. The E_2g_ mode at 130 cm^−1^

The Raman mode at 130 cm^−1^ is accredited to the In–O bond in the InO_6_ octahedral unit and is also indicative of the crystal quality of the films.^48^ It is noted that the intensity of the peak is maximum for the films annealed at 350 °C, then decreases for 400 °C, suggesting that annealing at 350 °C likely facilitates sufficient grain growth and minimizes structural defects without inducing significant thermal stress. These findings, supported by X-ray diffraction (XRD) analysis, collectively indicate that annealing at 350 °C establishes the most favourable conditions for achieving superior crystal quality of the thin films.

##### 3.1.2.2. The E_1g_ mode at 306 cm^−1^

The peak at 306 cm^−1^ demonstrates the bending vibrations of InO_6_ octahedra. This mode is sensitive to oxygen vacancies.^49^ Since the intensity of Raman scattering is directly related to the square of the derivative of the polarizability with respect to the normal mode amplitude, a change in intensity can be anticipated upon the formation of oxygen vacancies. The highest intensity of the peak at 350 °C annealing temperature indicates the maximum amount of oxygen vacancies which is further supported by PL results.

##### 3.1.2.3. The A_g_ mode at 561 cm^−1^

The Raman mode at 561 cm^−1^ demonstrates symmetric lattice vibrations of InO_6_ octahedra. The broadening of the Raman peak is observed for annealing temperature of 400 °C. Annealing at elevated temperatures can lead to non-stoichiometric oxygen deficiency and thermal stress, causing microstructural modifications such as grain boundary defects, cracks or strain, which contribute to the broadening of Raman peaks.^50^

#### 3.1.3. XPS analysis

X-ray photoelectron spectroscopy (XPS) was employed to investigate the elemental composition, and chemical states present in the In_2_O_3_ nanostructured films. The XPS survey spectra for samples annealed at 300 °C and 350 °C are shown in the Section 2 (Fig. 2) of ESI.† All spectra were calibrated using the C 1s peak at 284.4 eV as a reference. The survey scan revealed distinct photoemission peaks associated with various indium and oxygen states, including In 3s, In 3p, In 3d, In 4s, In 4p, In 4d, and O 1s, along with Auger peaks corresponding to In MNN and O KLL, and a C 1s signal. The characteristic binding energies (BE) observed were: 828 eV (In 3s), 703 eV (In 3p_1/2_), 665 eV (In 3p_3/2_), 452 eV (In 3d_3/2_), 444 eV (In 3d_5/2_), 123 eV (In 4s), 78 eV (In 4p), 17 eV (In 4d), and a broad In MNN peak in the range of 1076–1084 eV. For a more detailed understanding of the chemical environment, high-resolution scans of the In 3d and O 1s core levels were further analysed.

##### 3.1.3.1. In 3d core level spectra

**Fig. 5:**
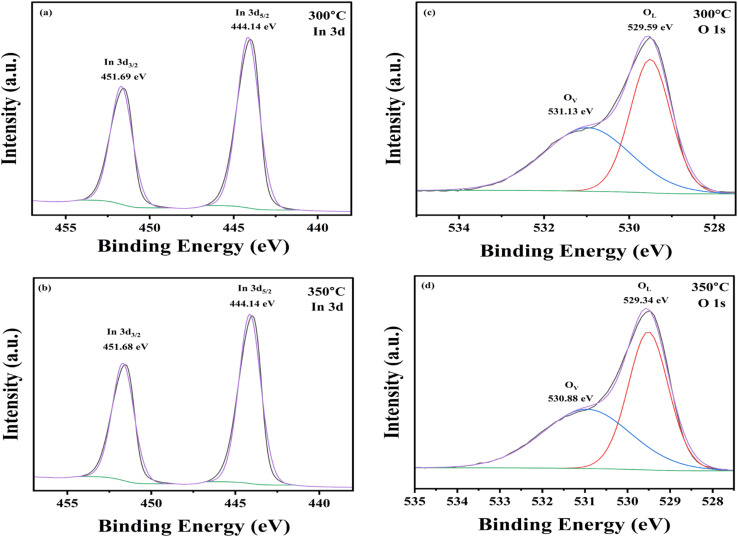
High-resolution XPS spectra of In_2_O_3_ thin films: (a) In 3d core level for the film annealed at 300 °C, (b) In 3d core level for the film annealed at 350 °C, (c) O 1s core level for the film annealed at 300 °C, and (d) O 1s core level for the film annealed at 350 °C.

Fig. 5(a) and (b) shows the core-level spectra of Gaussian-deconvoluted In_2_O_3_ thin films annealed at 300 °C and 350 °C. The spectra exhibit two characteristic In 3d components, namely In 3d_5/2_ and In 3d_3/2_. The binding energy maxima are observed at 444.1 eV and 451.7 eV, respectively, corresponding to the spin–orbit splitting of the In 3d levels. These peaks confirm the presence of trivalent indium ions (In^3+^) in the films. The energy separation between In 3d_5/2_ and In 3d_3/2_ is approximately 7.6 eV, which is consistent with values reported in the literature^51^

##### 3.1.3.2. O 1s core level spectra

The O 1s core-level spectra presented in Fig. 5(c) and (d) exhibit two well-resolved peaks, corresponding to lattice oxygen (O_L_) and oxygen vacancies (O_v_). For the film annealed at 300 °C, the peaks are centered at binding energies of 529.59 eV and 531.13 eV, while for the film annealed at 350 °C, the peaks appear slightly shifted to lower binding energies at 529.34 eV and 530.88 eV, respectively. Quantitative analysis based on peak area fitting reveals that the proportions of lattice oxygen and oxygen vacancies are 51.23% and 48.77% for the 300 °C sample, and 51.90% and 48.10% for the 350 °C sample. Although the relative amounts of oxygen vacancies remain consistent across the two annealing conditions, the observed shift towards lower binding energies after annealing at 350 °C suggests an enhancement in the structural order of the film. Improved crystallinity and reduced defect density facilitate more efficient charge transport and suppress electron–hole recombination. Consequently, the surface becomes more chemically active and accessible, thereby enhancing the material's potential for gas sensing applications.^52^

### 3.2. Morphology of annealed In_2_O_3_ nanostructures

**Fig. 6:**
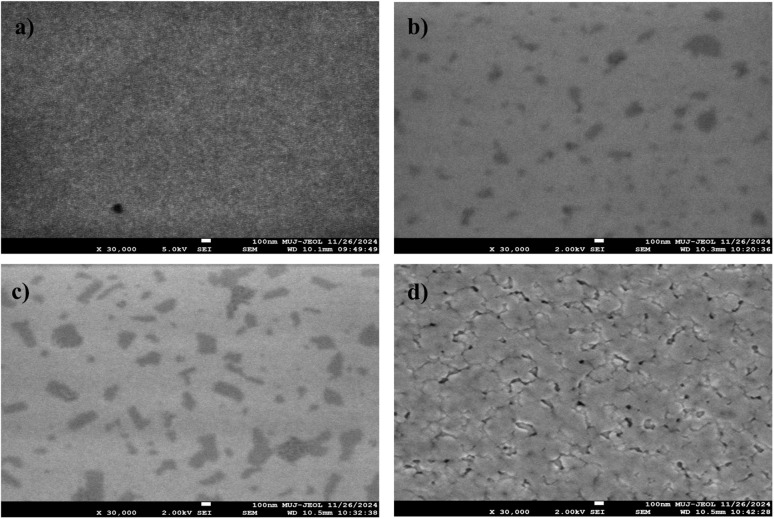
FESEM images of nanostructured indium oxide thin films for various annealing temperatures of (a) 250 °C, (b) 300 °C, (c) 350 °C and (d) 400 °C.

The thin films morphology was analysed using Field Emission Scanning Electron Microscopy (FESEM). The Fig. 6 presents FESEM descriptions of In_2_O_3_ thin films subjected to annealing at different temperatures.

The films annealed at 250 °C exhibit a smoother surface with smaller, uniformly distributed grains. Structural imperfections, such as defects or voids, are minimal at this temperature. However, with an increase in annealing temperature of 350 °C, there is a noticeable rise in defects and grain boundaries. The appearance of darker areas suggests an increase in voids and a higher concentration of oxygen vacancies. These vacancies serve as defect sites, contributing to greater porosity.^53^ This increase in defect density can be attributed to non-stoichiometric conditions caused by the partial loss of oxygen atoms from the lattice. The film annealed at 400 °C exhibits surface cracks, suggesting defects caused by thermal stress.^54^ These defects may compromise the mechanical stability of the film.

### 3.3. Optical studies

#### 3.3.1. Photoluminescence studies of In_2_O_3_ nanostructures

**Fig. 7:**
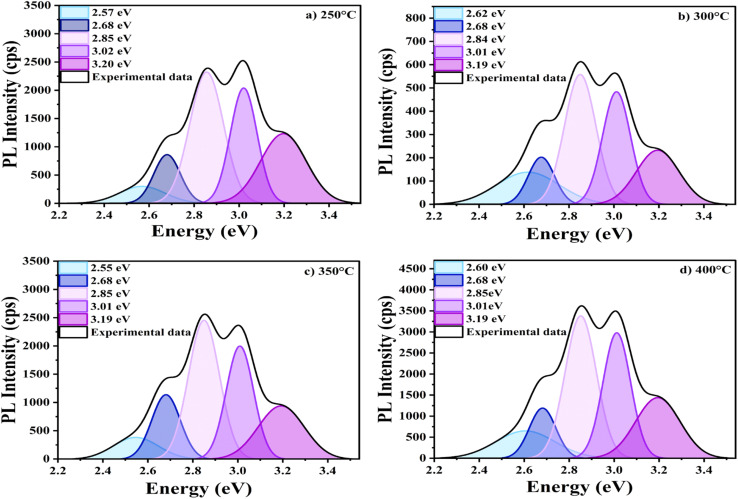
Gaussian deconvoluted PL emission spectra of In_2_O_3_ nanostructures annealed at (a) 250 °C, (b) 300 °C, (c) 350 °C, and (d) 400 °C temperatures.

The photoluminescence (PL) spectroscopy analysis, conducted with an excitation wavelength of 335 nm, reveals presence of defects in In_2_O_3_ thin films. Indium oxide thin films typically exhibit intrinsic defects such as antisites, vacancies, and interstitials of indium as well as oxygen. The PL spectra of In_2_O_3_ films, shown in Fig. 7, were analysed through Gaussian deconvolution. The parameters derived from this analysis are presented in Table 2, offering comprehensive details about the defect states responsible for the detected PL emissions. This method enables the precise identification and characterization of various defects by isolating the contributions of distinct emission centers.

**Table 2:** Deconvoluted PL peak parameters of In_2_O_3_ nanostructured thin films

Annealing temperature (°C)	Parameters	UV (3.19 eV)	Violet I (3.01 eV)	Violet II (2.85 eV)	Blue I (2.68 eV)	Blue II (2.55–2.62 eV)
250	Intensity	1232	2039	2320	859	303
	FWHM	0.24	0.14	0.18	0.14	0.24
300	Intensity	231	485	559	203	138
	FWHM	0.22	0.15	0.16	0.14	0.33
350	Intensity	940	1997	2458	1140	376
	FWHM	0.24	0.14	0.16	0.15	0.24
400	Intensity	1439	2980	3370	1187	657
	FWHM	0.23	0.14	0.16	0.14	0.3

For In_2_O_3_ thin films, the photoluminescence (PL) spectra reveal emissions that can be broadly categorized into a prominent ultraviolet (UV) or near-band-edge (NBE) emission band, along with significant deep-level emissions (DLEs). The NBE emission typically corresponds to higher energy transitions near the band gap, while the DLEs arise from energies associated with defect states within the band gap. The ultraviolet emission, with an energy of 3.20 eV, is attributed to the recombination of free excitons, which may occur *via* exciton–exciton collisions. The near-band-edge (NBE) emission peak (3.20 eV) appeared weaker when compared to some of the peaks from deep-level emission. Such behaviour suggests that the relaxation of photoexcited carriers from the conduction band to defect states occurs more rapidly than their recombination with valence band holes.^55^ Saeed *et al.* associated the weak NBE emission with the occurrence of defects, including oxygen vacancies. They noted that a high ratio of NBE to deep-level emission is typically accredited to the quantum confinement effect. However, in this case, the contribution of quantum confinement effect to the observed photoluminescence can be ruled out.^56^ On the other hand, DLE bands, observed at energies of 2.57–3.02 eV, are generally linked to intrinsic point defects, including oxygen vacancies, interstitials, or antisites, within the In_2_O_3_ films.^57^ These visible emissions are believed to result from the recombination of deeply trapped electrons with delocalized holes in the valence band or from delocalized electrons recombining with deeply trapped holes in the conduction band. The violet-blue emission peaks observed around 3.02 eV, 2.85 eV, and 2.68 eV could be attributed to indium–oxygen vacancy centres and oxygen vacancies. The broad blue emission peak centered at 2.57 eV could be attributed to indium interstitials.^58–60^

Fig. 7 illustrates how annealing temperature influences the position and intensity of defect-related peaks. The peak positions remain unchanged as the annealing temperature increases, but the intensity is lowest at 300 °C. The reduction in photoluminescence (PL) intensity is attributed to a reduction in oxygen vacancies. A similar trend was reported by W. Zhong *et al.*,^61^ where the PL intensity decreased at 300 °C due to a reduction in defect density. This occurs as oxygen atoms fill the vacancies, resulting in films with improved stoichiometry. This behaviour is commonly observed in many materials, where the incorporation of oxygen reduces the density of defects, leading to diminished luminescent properties. When the annealing temperature increases from 350 °C to 400 °C, the PL intensity increases again. This can be explained by the desorption of oxygen at elevated temperatures, which creates more oxygen vacancies and defects in the film. For ITZO films, a notable increase in PL intensity was observed due to the development of non-stoichiometric conditions resulting from the loss of lattice oxygen.^61^ A similar trend has been reported for ZnO thin films, where higher annealing temperatures lead to an increase in defect density, thereby enhancing the PL intensity.^62^

No significant changes are observed in the FWHM of PL emission spectrum. This means that the nature of the emission remains the same, even though the efficiency of the emission changes.

### 3.4. Analysis of In_2_O_3_ nanostructures for H_2_S gas sensing

Studies on In_2_O_3_-based sensors have been carried out for various gases; however, the detection of H_2_S is particularly important due to its highly toxic and corrosive properties, even at low concentrations. The significant risks H_2_S poses to human health and the environment, highlight the need for precise and reliable detection, making it a crucial target for gas sensing applications. Consequently, this study is focused specifically on the detection of H_2_S gas.

**Fig. 8:**
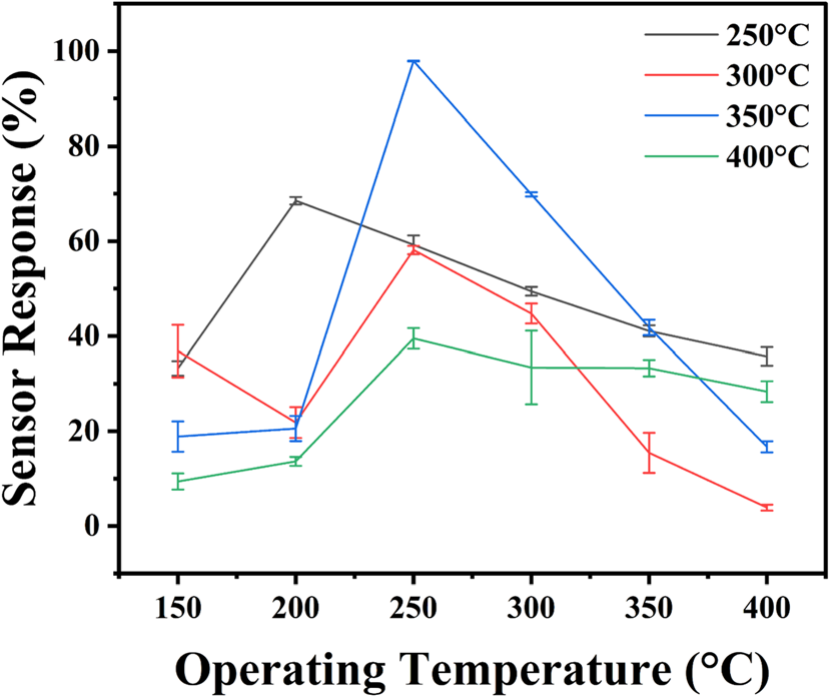
Operating temperature studies for nanostructured indium oxide sensors annealed at various temperatures.

The operating temperature is a key feature in evaluating the efficiency of MOS-based gas sensors, as it significantly influences gas adsorption, desorption, and surface interactions. To identify the optimal operating temperature, In_2_O_3_ nanostructured gas sensors were tested for their response towards 4 ppm H_2_S across a temperature range of 150–400 °C, as illustrated in Fig. 8. The sensor response *versus* operating temperature studies were performed for all the sensors over three cycles to ensure reproducibility and reliability. The mean and standard deviation of the sensor responses were calculated and are presented in the Fig. 8, as well as in the ESI Table TS2.† The statistical analysis revealed that the level of significance for the observed trends is <0.05, confirming the robustness of the results.

The results indicate that the optimum operating temperature is 250 °C. The sensors' responses to H_2_S steadily increase with temperature from 150 °C to 250 °C. Below 250 °C, H_2_S gas molecules necessitate nominal energy to surpass the threshold activation energy and interact with adsorbed oxygen species. However, above 250 °C, the responses begin to diminish. This reduction occurs because, at higher temperatures, the capacity for gas adsorption weakens, leading to rapid desorption before surface reactions can take place.^63^ The consistent performance across multiple cycles, supported by statistical significance, underscores the reliability of the identified optimal temperature.

The gas sensing mechanism of In_2_O_3_ towards H_2_S primarily operates on a chemiresistive principle, wherein the electrical resistance of the sensor varies with the concentration of the target gas.^64^ In_2_O_3_, being an n-type semiconductor, exhibits resistance changes due to surface redox reactions. When the sensor is exposed to air at elevated temperatures, oxygen molecules are adsorbed onto the surface and extract electrons from the conduction band of In_2_O_3_, forming chemisorbed oxygen species such as O_2_− and O_2_2−. This results in the formation of an electron depletion layer at the surface, which increases the baseline resistance of the sensor. The reactions involved in oxygen chemisorption can be summarized as follows:^30^

3.5 O_2_(g) + e^−^ ⇌ O_2_−(ad)

3.6 O_2_^−^(ad) + e^−^ ⇌ O_2_^2−^(ad)

Upon exposure to H_2_S, a reducing gas, it interacts with these chemisorbed oxygen species on the sensor surface, donating electrons back to the In_2_O_3_ conduction band. This electron release reduces the sensor's resistance and restores charge carriers, enhancing the conductivity. The primary reactions during this process are:^30^

3.7 H_2_S(g) + e^−^ ⇌ H_2_S(ad)

3.8 H_2_S(g) + O_2_x−(ad) ⇌ H_2_S(ad) + O_2_(g) + *x*e^−^

3.9 2H_2_S(ad) + 3O_2_x−(ad) ⇌ 2SO_2_(g) + 2H_2_O + 3*x*e^−^

**Fig. 9:**
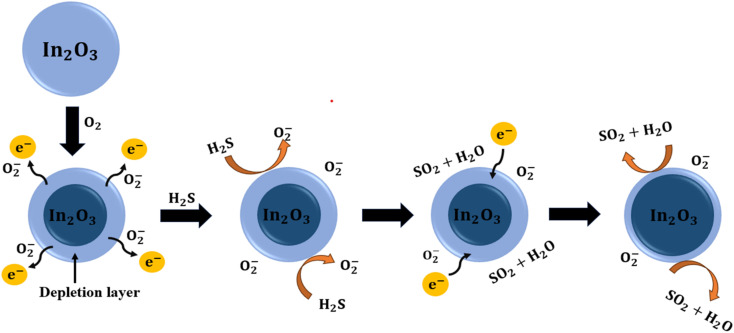
Schematic diagram representing the H_2_S gas sensing mechanism for indium oxide nanostructured sensors.

**Fig. 10:**
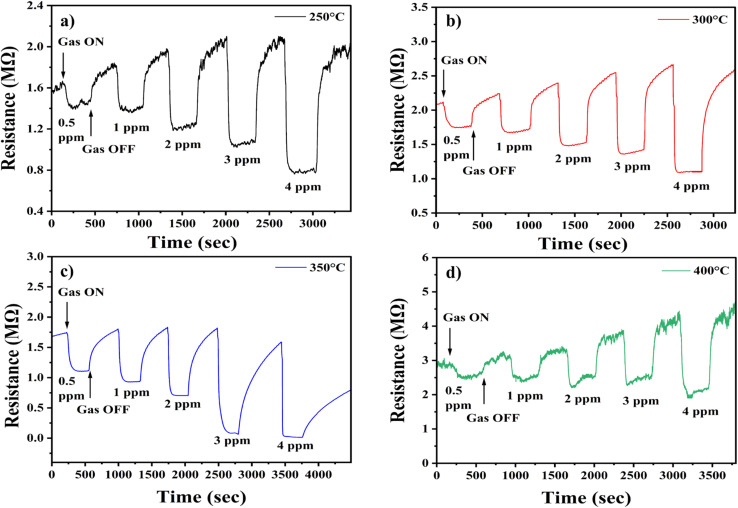
Sensor response curves for In_2_O_3_ gas sensors annealed at (a) 250 °C, (b) 300 °C, (c) 350 °C, and (d) 400 °C temperatures.

Once the H_2_S flow is stopped and synthetic air is reintroduced, oxygen molecules re-adsorb onto the surface, recapturing electrons and restoring the depletion region.^24^ The dynamic sensing behaviour and thermal activation process are schematically illustrated in Fig. 9. Additionally, the response trends observed in Fig. 10 demonstrate that sensor performance improves with increasing H_2_S concentration, which can be attributed to enhanced adsorption kinetics and more effective surface reactions at higher analyte levels.^65^ The response and recovery parameters were calculated as detailed in the Experimental section. These findings collectively support the role of surface chemistry, oxygen vacancy dynamics, and thermally driven kinetics in enabling sensitive and selective H_2_S detection using In_2_O_3_ – based sensors.^23^

The data presented in ESI (TS3†) highlights the performance of the In_2_O_3_ sensors in terms of response time and recovery time at various concentrations of H_2_S gas and annealing temperatures, with an optimized operating temperature of 250 °C. The results clearly indicate that the response time is consistently faster than the recovery time. This phenomenon can be attributed to the faster adsorption and reduction reactions of H_2_S gas on the sensor surface compared to the slower desorption and oxidation reactions that occur during recovery. This trend is consistent with previous studies.^66^

**Fig. 11:**
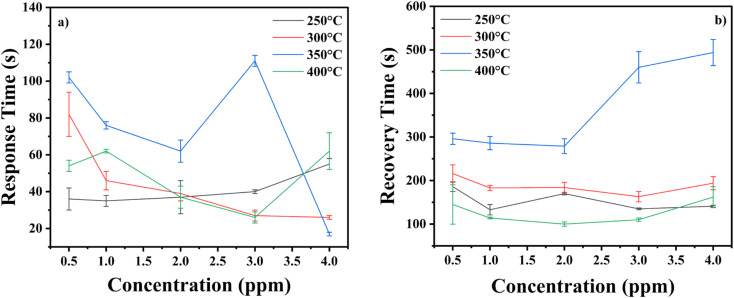
(a) Response time plots (b) recovery time plots for In_2_O_3_ sensors annealed at 250 °C, 300 °C, 350 °C and 400 °C for H_2_S gas.

At higher concentrations of H_2_S gas, the sensor exhibits quicker response times. For instance, at 4 ppm of H_2_S gas, the sensor annealed at 350 °C demonstrates the fastest response time of 17 ± 1 seconds. These observations are further supported by Fig. 11, which illustrates the response and recovery times as functions of H_2_S gas concentrations and annealing temperatures. The graph shows that the sensor annealed at 350 °C outperforms others in terms of response time while its recovery is slowest. Additionally, the graph includes error bars representing the standard deviation, which were calculated from three consecutive measurement cycles for each condition. The detailed summary of the mean values and standard deviations have been provided in ESI (Table S4†).

To ensure the statistical significance of the results, a level of significance (*p*-value) was calculated for the data. The *p*-value was found to be less than 0.05, indicating that the observed differences in response and recovery times, are statistically significant and not due to random variation. This reinforces the reliability of the findings and underscores the impact of annealing temperature and gas concentration on the sensor's performance.

**Fig. 12:**
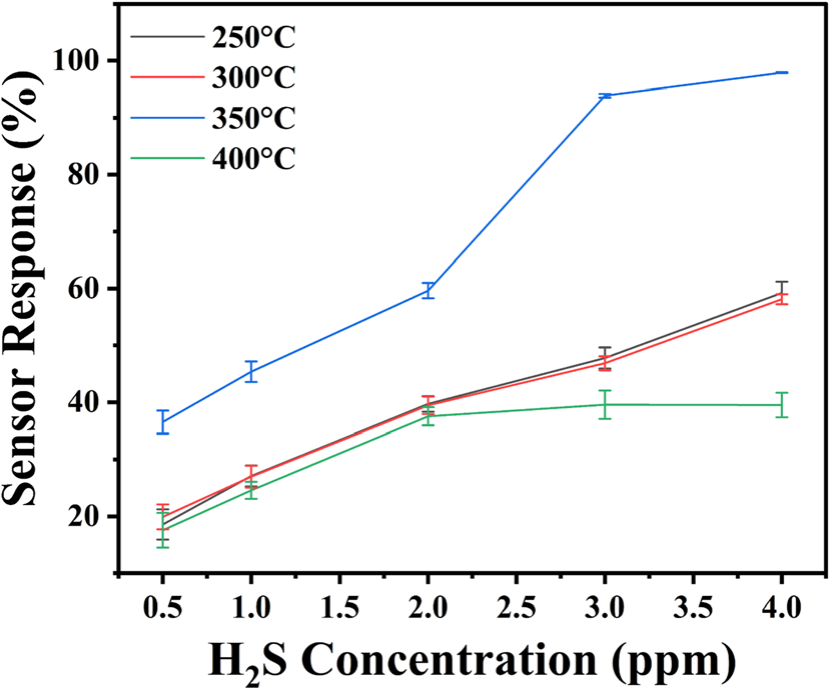
Sensor response values for different concentrations of H_2_S gas.

To investigate the sensor response of the proposed H_2_S sensor at its optimum operating temperature, the transient sensor response was evaluated for H_2_S gas concentrations ranging from 0.5 to 4 ppm, as illustrated in Fig. 12. For a detailed analysis, the mean and standard deviations of the sensor response were also calculated using three consecutive measurement cycles. These standard deviations are represented as error bars in the graph providing a visual representation of the variability in the sensor response across the measurement cycles. The statistical significance of the results was also evaluated, and the *p*-value was found to be less than 0.05, confirming that the observed differences in sensor response are statistically significant. A comprehensive table summarizing these findings, including the mean, standard deviations, and level of significance, is provided in the ESI TS4† for further reference. The sensor annealed at 350 °C exhibited the highest sensor response value of (97.89 ± 0.08)%, highlighting its superior sensitivity at this annealing temperature. However, when the annealing temperature was increased to 400 °C, the sensor's response decreased significantly across all tested gas concentrations, suggesting that higher annealing temperatures may negatively impact the sensor's performance. Further, the results demonstrate a strong linear correlation between the sensor response and H_2_S concentration, with a lower limit of detection of 0.5 ppm. This indicates that the In_2_O_3_-based sensor is capable of quantitatively detecting H_2_S in air with high precision.

The H_2_S gas sensing behaviour of In_2_O_3_ thin films is influenced by various aspects, including defects, crystallinity, grain boundaries, and morphology of the films.^30^ As outlined in the gas sensing mechanism, oxygen vacancy (*V*_O_) defects play a pivotal role by acting as the primary adsorption sites for H_2_S gas, enabling effective detection.^64^

The oxygen-deficient materials, which provide a substantial number of contact sites, are thoroughly linked to the mechanism of gas sensing, facilitating significant changes in the electrical resistance.^67^ The rise in oxygen vacancy defects could be accountable for the notable improvement in the response of In_2_O_3_ thin films to the lower H_2_S gas concentrations. Based on the Raman and PL study, it is concluded that the sensor annealed at 350 °C has a higher concentration of oxygen vacancies compared to the other films, which aligns with its enhanced sensing response. W. Chen *et al.*^68^ reported that the increase in the number of oxygen vacancies after adding dopant provides donor states to In_2_O_3_ NBs, improves its electrical conductivity and thus, enhances its sensor response. J. Liu *et al.*^69^ demonstrated the significant role of oxygen vacancy defects in enhancing the sensitivity to H_2_S gas. M. Kaur *et al.*^49^ stated that the uptake and release of target gas is influenced by the interaction between bond energy of metal and oxygen, oxygen vacancies, and surface oxygen. They identified oxygen vacancies (*V*_O_) as crucial active sites for adsorption, playing a noteworthy role in the gas sensing mechanism. Although the film annealed at 400 °C exhibits the highest PL intensity, its sensing performance is not optimal. The decrease in sensor response could be due to the thermal stress and presence of cracks as confirmed from FESEM, making some active sites inaccessible for the target gas to interact. Moreover, for the films annealed at 350 °C, voids are visible from FESEM results, which may contribute to the enhanced gas sensing performance by providing higher surface area for gas adsorption. Numerous studies have highlighted the significant potential of materials with high surface area for effectively adsorbing target gas molecules.^70–72^

Additionally, XPS analysis further supports these findings where the shift towards lower binding energies after annealing at 350 °C suggests improved structural order, enhanced crystallinity, and reduced defect density thus facilitating more efficient charge transport, suppress electron–hole recombination, and render surface more chemically active and accessible, thereby significantly boosting the gas sensing performance.

**Fig. 13:**
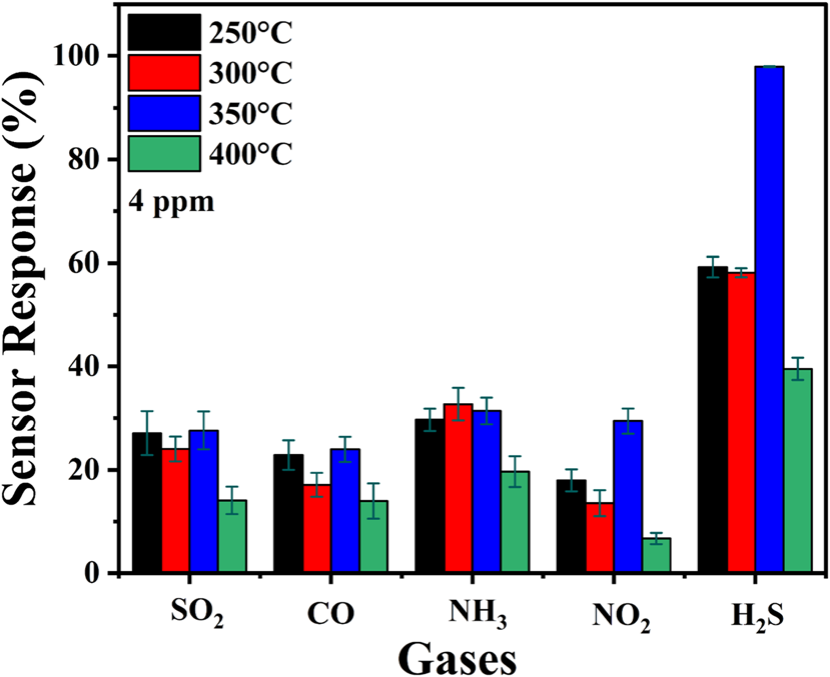
Selectivity histogram of H_2_S with SO_2_, CO, NH_3_ and NO_2_ gases.

The ability of In_2_O_3_ sensors to specifically detect H_2_S is critical for gas sensing applications. Selectivity, defined as the capability of sensor to differentiate a target gas with respect to other gases in a mixture, is a key parameter in this context. In this study, the sensors were evaluated against several gases, which include SO_2_, CO, NH_3_, NO_2_, and H_2_S. The selectivity data of the proposed H_2_S sensor is visualized in a histogram as shown in Fig. 13. The histogram provides a clear comparison of the sensor's response to H_2_S gas relative to other interfering gases, demonstrating its high selectivity for H_2_S. Error bars are included in the plot to represent the standard deviations calculated from three consecutive measurement cycles. These error bars highlight the variability in the sensor's response and further validate the consistency of its performance.

The corresponding values for selectivity, including the mean response and standard deviations for each gas, are tabulated in TS5 of the ESI.† This table provides a detailed breakdown of the sensor's response to various gases, complementing the graphical representation in Fig. 13. Among the tested sensors, the one annealed at 350 °C demonstrated excellent selectivity for H_2_S, making it particularly suitable for detecting this gas.

The long-term stability of the In_2_O_3_ – based gas sensor annealed at 350 °C was evaluated after eight months of storage under ambient conditions. The dynamic response curves of the freshly fabricated and aged sensors are presented in the ESI (Fig. S3†). During the long-term measurements, the relative humidity (RH) inside the sealed gas sensing chamber was monitored using a DHT11 humidity sensor with an accuracy of ±5% RH. Initially, the RH was 73% under ambient conditions but decreased to 17% during sensor operation at 250 °C, primarily due to water desorption at elevated temperature.

A noticeable decrease in the baseline resistance of the aged sensor was observed compared to the fresh one. This change is attributed to the influence of humidity, which plays a significant role in the aging behaviour of oxide-based sensors. Over time, adsorbed water molecules interact with lattice oxygen ions in In_2_O_3_, generating free electrons and reducing baseline resistance.^73^ Despite this shift, the aged sensor exhibited only a slight (∼23%) decline in response to 0.5 ppm H_2_S gas, confirming its good long-term stability.^74^ Furthermore, the calculated sensor response values for both the fresh and aged sensors remained relatively consistent, indicating that the low-humidity environment during testing had minimal impact on sensor performance. These findings highlight the sensor's reliable performance over extended periods, with minor degradation likely influenced by environmental humidity and natural aging processes.

Table 3 presents a comparison of the prepared In_2_O_3_ H_2_S sensor along with recently reported metal oxide H_2_S sensors. In this work, the sensor demonstrated a remarkable response towards the lowest limit of detection (LOD) of 0.5 ppm H_2_S gas. Additionally, the reported In_2_O_3_ based H_2_S sensor exhibited good sensing performance at a concentration of 4 ppm. The findings from Raman analysis, photoluminescence spectroscopy, and FESEM analysis indicate that an increase in oxygen vacancies and surface defects confirms the influence of annealing temperature on improving H_2_S gas sensing response.

**Table 3:** Comparison of indium oxide sensor for H_2_S gas sensing with other reported metal oxide H_2_S gas sensors

Sensing material	Operating temperature (°C)	Concentration (ppm)	Sensor response	Response/recovery time (s)	References
In_2_O_3_	250	4	99.14%	20/606	Present work
CuO/SnO_2_	200	50	85.71%	100/109	12
NiO	400	200	28.8	108/47	14
In_2_O_3_	RT	5	68%	18/507	66
In_2_O_3_/ZnO	250	50	44.5	24/27	71
Nd_2_O_3_-loaded In_2_O_3_	300	10	10.11	—	75
Ag/WO_3_/rGO	150	100	685.8	8/38	76

## 4. Conclusions

In_2_O_3_ thin films were synthesized using a chemical route spin-coating method. Their properties were systematically analysed through XRD, Raman, XPS, UV-vis spectroscopy, photoluminescence, and FESEM. The sensors were tested for H_2_S gas sensing performance at small concentrations ranging from 0.5 to 4 ppm. Among the samples, the sensor annealed at 350 °C demonstrated a fast response time and significantly enhanced sensor response within the tested range, achieving optimal performance at an operating temperature of 250 °C. The improved sensing characteristics were primarily accredited to the existence of voids and oxygen vacancies, which facilitated gas adsorption. This study highlights the successful optimization of annealing temperature to develop In_2_O_3_ thin films for effective, and selective H_2_S gas detection applications. However, this study has notable limitations. One notable drawback is the relatively long recovery time observed in the sensor's performance. This delay in recovery can be attributed to the slower desorption and oxidation processes of H_2_S molecules from the sensor surface, which limits its efficiency in real-time applications where rapid cycling between gas exposure and recovery is essential. Addressing this limitation will be crucial for improving the practicality of the sensor in real-world scenarios.

Another limitation concerns the long-term stability of the sensor. Although the sensor showed reliable performance during testing, a noticeable decrease in baseline resistance was observed in aged sensors after eight months of storage, primarily due to environmental humidity and natural aging processes. Despite this, the sensor still exhibited good stability, with only a slight (∼23%) decrease in response to 0.5 ppm H_2_S, indicating its robustness. However, improving the sensor's resistance to humidity and aging will be essential for extending its lifespan and enhancing its reliability in long-term applications.

To overcome the current limitations and further enhance the performance of In_2_O_3_-based H_2_S sensors, future studies could focus on several key areas. First, the incorporation of catalytic additives or dopants into the In_2_O_3_ matrix could be explored to accelerate the desorption process and reduce recovery time. Second, the development of hybrid nanostructures or composite materials, combining In_2_O_3_ with rGO or other metal oxides, may improve both response and recovery characteristics while maintaining high selectivity.

## Data availability

The data cannot be made publicly available upon publication as they are not available in a standard format that is sufficiently accessible by other researchers. The data that support the outcomes of this study will be shared upon reasonable request from the authors.

## Conflicts of interest

There are no conflicts to declare.

## Supplementary Material

RA-015-D5RA01394A-s001
