# Supplementary material for: Optimized nanostructured In2O3 gas sensors: harnessing annealing-induced defects and oxygen vacancies for ultra-sensitive and selective H2S detection at trace levels
Source: RSC Adv. 2025 May 19;15(21):16555–69. doi: 10.1039/d5ra01394a (PMC12086529; doi:10.1039/d5ra01394a)
Supplement: RA-015-D5RA01394A-s001 [file RA-015-D5RA01394A-s001.pdf]

Supplementary Information (SI) for RSC Advances.
This journal is © The Royal Society of Chemistry 2025

Supporting Information

Optimized Nanostructured In_2O_3 Gas Sensors: Harnessing Annealing- Induced Defects and Oxygen Vacancies for Ultra-Sensitive and Selective H_2S Detection at Trace Levels

Tanya Sood[1], Ramseena Thundiyil[1], Anusha[1], Saikat Chattopadhyay[2], P. Poornesh[1*]

[1]Department of Physics, Manipal Institute of Technology, Manipal Academy of Higher Education, Manipal 576104, India

[2]Department of Physics, School of Basic Sciences, Manipal University Jaipur, Jaipur 303007, India

[*]Corresponding author: poornesh.p@manipal.edu, poorneshp@gmail.com

1. UV-Visible spectroscopy of In_2O_3 nanostructures:

Examining the optical properties of semiconducting materials is essential as it provides insights into their band structures and the nature of electronic transitions. Fig. 1 (a) illustrates the optical transmittance of In_2O_3 nanostructures annealed at several annealing temperatures across the spectral range of 300-1100 nm. The thin films exhibit excellent transparency. The transmittance oscillations observed in the 400–1100 nm range indicate that the films have smooth interfaces. The reduction in transmittance at higher annealing temperatures may be accredited to an increase in the number of defects, which scatter light and reduce transmittance [1].

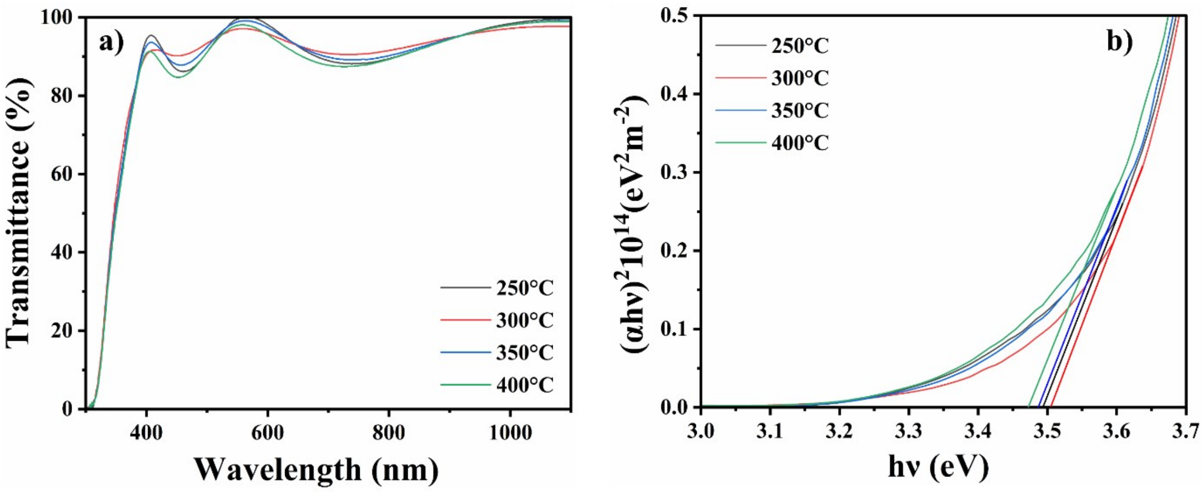

Fig. 1 a) Transmittance spectra and b) Tauc's plot of In_2O_3 nanostructured films for various annealing temperatures

Tauc's relation is utilized to determine the energy bandgap (E_g) values, expressed as [2],

$$\alpha h\nu = B(h\nu - E_g) \quad (3.5)$$

In the equation, α denotes the absorption coefficient, hv denotes photon energy, E_g indicates the optical bandgap, while B is a constant that depends on the transition probability. The exponent n defines the type of the transition. In Tauc's plot, $h\nu$ is plotted on x-axis and $(\alpha h\nu)^{1/n}$ is plotted on the y-axis. For direct allowed transitions, n = ½, while for indirect allowed transitions, n = 2. The absorption coefficient (α) can be determined utilizing the Beer-Lamberts law [3],

$$\alpha = \frac{1}{t}\ln\left(\frac{1}{T}\right) \quad (3.6)$$

Where t denotes the obtained film's thickness and T represents the transmittance obtained by the films.

Fig. 1 b) illustrates the Tauc's plot for direct allowed transitions of In_2O_3 nanostructures for various annealing temperatures and the band gap values are listed in. The band gap values of In_2O_3 films fall well within the standard range and is given in the Table 1.

Table 1 Band gap energy (Eg) of indium oxide nanostructured thin films

**Annealing Temperature (°C)**	**Band gap energy, E_g (eV)**
250	3.49
300	3.50
350	3.49
400	3.47

2. XPS Analysis:

X-ray photoelectron spectroscopy (XPS) was employed to investigate the elemental composition, and chemical states present in the In_2O_3 nanostructured films. The XPS survey spectra for samples annealed at 300 °C and 350 °C are shown in the Fig. 2. All spectra were calibrated using the C 1s peak at 284.4 eV as a reference. The survey scan revealed distinct photoemission peaks associated with various indium and oxygen states, including In 3s, In 3p, In 3d, In 4s, In 4p, In 4d, and O 1s, along with Auger peaks corresponding to In MNN and O KLL, and a C 1s signal. The characteristic binding energies (BE) observed were: 828 eV (In 3s), 703 eV (In $3p_{1/2}$), 665 eV (In $3p_{3/2}$), 452 eV (In $3d_{3/2}$), 444 eV (In $3d_{5/2}$), 123 eV (In 4s), 78 eV (In 4p), 17 eV (In 4d), and a broad In MNN peak in the range of 1076–1084 eV.

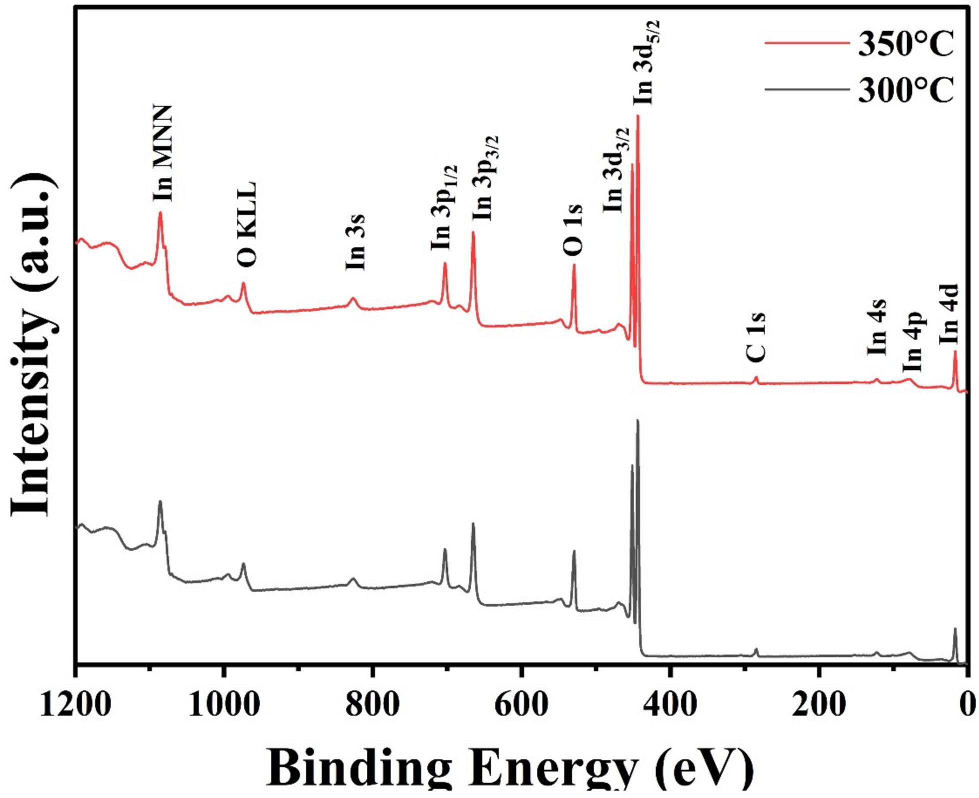

Fig. 2 XPS survey spectra of In_2O_3 films annealed at 300°C and 350°C

3. Analysis of In_2O_3 nanostructures for H_2S gas sensing:

Table 2 Operating temperature studies for nanostructured indium oxide sensors annealed at various temperatures for 4 ppm of H_2S gas

Annealing Temperature (°C)	Operating Temperature (°C)	Sensor Response (%)			Mean ± Standard Deviation
		Cycle 1	Cycle 2	Cycle 3	
250	150	31.84	32.99	34.87	33.23 ± 1.53
	200	67.66	68.65	69.25	68.52 ± 0.80
	250	57.24	59.24	61.19	59.22 ± 1.98
	300	48.47	49.46	50.34	49.42 ± 0.93
	350	39.97	41.00	42.28	41.08 ± 1.16
	400	33.99	35.40	37.89	35.74 ± 1.94
300	150	31.02	37.49	42.02	36.84 ± 5.52
	200	18.45	21.99	24.99	21.81 ± 3.27
	250	57.33	58.01	59.06	58.13 ± 0.87

	300	43.91	43.16	47.14	44.73 ± 2.11
	350	12.43	13.58	20.26	15.42 ± 4.22
	400	3.22	3.91	4.48	3.872 ± 0.63
350	150	15.49	19.29	21.82	18.87 ± 3.19
	200	20.14	18.13	23.39	20.55 ± 2.65
	250	97.80	97.91	97.96	97.89 ± 0.08
	300	70.28	69.92	69.41	69.87 ± 0.44
	350	40.10	41.90	43.36	41.81 ± 1.63
	400	17.85	16.77	15.48	16.70 ± 1.18
400	150	7.48	9.90	10.76	9.38 ± 1.70
	200	12.56	13.87	14.43	13.62 ± 0.95
	250	37.24	39.75	41.54	39.51 ± 2.16
	300	24.56	36.59	39.01	33.39 ± 7.74
	350	31.50	33.31	34.94	33.25 ± 1.72
	400	26.10	28.31	30.54	28.32 ± 2.21

The values differ significantly at 95 % confidence level ($p < 0.05$).

Table 3 Response time, recovery time of indium oxide sensors towards different H2S gas concentrations

**Annealing Temperature (°C)**	**Gas Concentration (ppm)**	**Response Time (s)**			**Mean ± Standard Deviation**	**Recovery Time (s)**			**Mean ± Standard Deviation**
		***C1**	***C2**	***C3**		***C1**	***C2**	***C3**	
250	0.5	35	43	31	36 ± 6	186	197	174	186 ± 11
	1	31	38	35	35 ± 3	119	140	141	133 ± 12
	2	46	36	28	37 ± 9	171	167	173	170 ± 3
	3	41	39	40	40 ± 1	133	136	137	135 ± 2
	4	52	57	56	55 ± 3	139	142	143	141 ± 2
300	0.5	71	80	94	82 ± 12	225	193	230	216 ± 20
	1	51	47	41	46 ± 5	179	190	180	183 ± 6
	2	40	42	35	39 ± 4	170	190	192	184 ± 12
	3	30	27	24	27 ± 3	160	177	153	163 ± 12

	4	27	26	25	26 ± 1	181	191	211	194 ± 15
350	0.5	99	103	104	102 ± 3	297	282	309	296 ± 13
	1	76	73	78	76 ± 2	270	290	299	286 ± 15
	2	56	61	69	62 ± 7	261	281	295	279 ± 17
	3	110	109	114	111 ± 3	419	485	477	460 ± 36
	4	16	17	18	17 ± 1	461	506	517	494 ± 30
400	0.5	50	54	57	54 ± 3	115	124	198	145 ± 45
	1	62	60	63	62 ± 1	116	112	115	114 ± 2
	2	31	38	43	37 ± 6	106	99	96	100 ± 5
	3	30	25	24	26 ± 3	108	114	107	110 ± 4
	4	71	64	51	62 ± 10	160	187	139	162 ± 24

*C1, C2 and C3 represents cycle 1, cycle 2 and cycle 3

The values differ significantly at 95 % confidence level ($p < 0.05$).

Table 4 Sensor Response values for different concentrations of H_2S gas for In_2O_3 gas sensors annealed at various temperatures

**Annealing Temperature (°C)**	**Gas Concentration (ppm)**	**Sensor Response (%)**			**Mean ± Standard Deviation**
		**Cycle 1**	**Cycle 2**	**Cycle 3**	
250	0.5	15.90	18.56	21.23	18.57 ± 2.66
	1	25.09	27.50	28.67	27.09 ± 1.82
	2	38.28	39.84	40.99	39.70 ± 1.36
	3	45.80	48.01	49.53	47.78 ± 1.87
	4	57.23	59.24	61.19	59.22 ± 1.97
300	0.5	17.51	20.44	21.79	19.91 ± 2.18
	1	25.01	27.03	28.87	26.97 ± 1.93
	2	37.83	39.71	40.85	39.4 ± 1.52
	3	45.62	46.85	48.10	46.86 ± 1.24
	4	57.33	58.01	59.06	58.1 ± 0.86
350	0.5	34.49	36.54	38.54	36.52 ± 2.02
	1	43.54	45.39	47.15	45.36 ± 1.80

	2	58.16	59.95	60.79	59.63 ± 1.34
	3	93.54	93.93	94.19	93.89 ± 0.32
	4	97.80	97.91	97.96	97.89 ± 0.08
400	0.5	14.30	17.97	20.38	17.55 ± 3.06
	1	23.13	24.48	26.13	24.58 ± 1.49
	2	35.90	37.60	39.12	37.54 ± 1.60
	3	37.05	39.62	42.01	39.56 ± 2.48
	4	37.24	39.75	41.54	39.51 ± 2.16

The values differ significantly at 95 % confidence level ($p < 0.05$).

Table 5 Selectivity studies of H_2S with SO_2, CO, NH_3 and NO_2 gases for In_2O_3 gas sensors annealed at various temperatures

**Annealing Temperature (°C)**	**Gas**	**Sensor Response**			**Mean ± Standard Deviation**
		**Cycle 1**	**Cycle 2**	**Cycle 3**	
250	SO_2	22.4	28.46	30.46	27.11 ± 4.20
	CO	20.13	22.71	25.85	22.90 ± 2.86
	NH_3	27.68	29.48	31.88	29.68 ± 2.11
	NO_2	15.97	17.78	20.22	17.99 ± 2.13
	H_2S	57.23	59.24	61.19	59.22 ± 1.97
300	SO_2	21.7007	24.037	26.498	24.07 ± 2.39
	CO	14.912	16.902	19.559	17.12 ± 2.33
	NH_3	29.572	32.601	35.86	32.67 ± 3.14
	NO_2	11.022	13.633	16.034	13.56 ± 2.56
	H_2S	57.338	58.012	59.062	58.13 ± 0.86
350	SO_2	23.829	28.009	31.062	27.63 ± 3.63
	CO	21.42	24.323	26.263	24.00 ± 2.43
	NH_3	28.661	31.634	33.809	31.36 ± 2.58
	NO_2	26.981	29.544	31.775	29.43 ± 2.39
	H_2S	97.806	97.914	97.969	97.89 ± 0.08
400	SO_2	11.853	13.403	17.031	14.09 ± 2.65
	CO	10.348	14.391	17.175	13.97 ± 3.43
	NH_3	16.478	20.172	22.392	19.68 ±2.98

	NO_2	5.687	6.59	7.882	6.71 ± 1.10
	H_2S	37.241	39.753	41.542	39.51 ± 2.16

The values differ significantly at 95 % confidence level ($p < 0.05$).

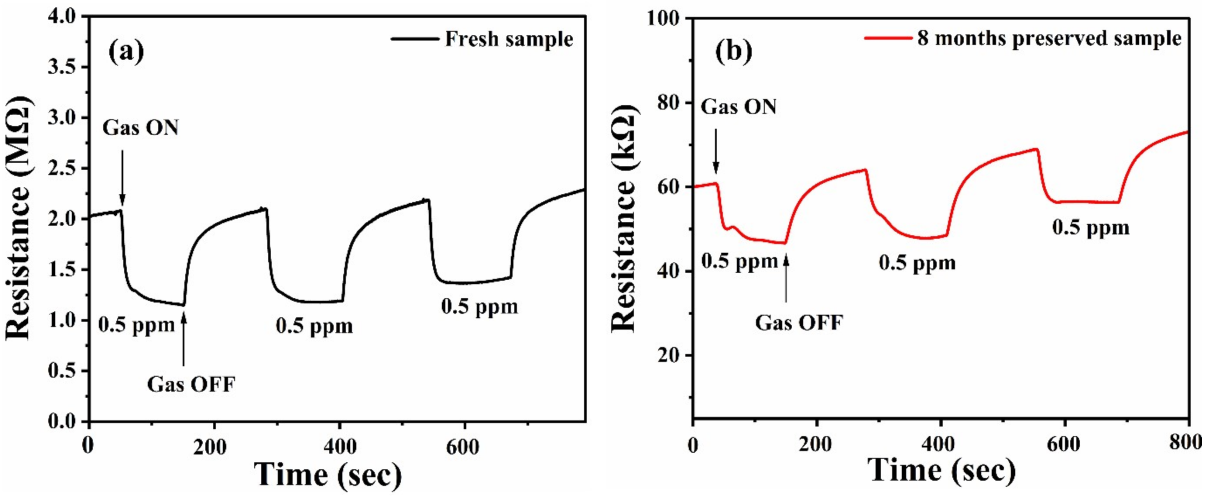

Fig. 3 Dynamic gas response of the sensor annealed at 350°**C**. (a) Response of the freshly prepared sensor subjected to three cycles of 0.5 ppm gas exposure. (b) Response of the same sensor after 8 months, highlighting its long-term stability

References

1 A. F. Al Naim, A. Solieman and E. R. Shaaban, 2022, 18, 373–388.

2 R. Rusdi, A. A. Rahman, N. S. Mohamed, N. Kamarudin and N. Kamarulzaman, *Powder Technol*, 2011, 210, 18–22.

3 M. Jothibas, C. Manoharan, S. Dhanapandian and S. J. Jeyakumar, *Asian Journal of Chemistry*.
